# 1252. A Real-World Observational Study on HIV-Infected Patients who Switched from Nevirapine + 2 Nucleoside Reverse Transcriptase Inhibitors to Dolutegravir/Lamivudine in British Columbia, Canada

**DOI:** 10.1093/ofid/ofac492.1083

**Published:** 2022-12-15

**Authors:** Joss de Wet, Joann K Ban, Gustavo Verdier, JueJing Ling, Maria Eberg, Andrean Bunko, Michael McKimm

**Affiliations:** University of British Columbia, Vancouver, British Columbia, Canada; GlaxoSmithKline Canada Inc., Mississauga, Ontario, Canada; ViiV Healthcare, Montréal, QC, Canada, Pointe-Claire, Quebec, Canada; IQVIA Solutions Canada Inc., Toronto, Ontario, Canada; IQVIA, Kirkland, Quebec, Canada; IQVIA Solutions Canada Inc., Toronto, Ontario, Canada; ViiV Healthcare, North Vancouver, British Columbia, Canada

## Abstract

**Background:**

Although convenient single-tablet antiretroviral regimens have been developed to treat HIV in recent years, some patients have continued to take a multi-tablet treatment, nevirapine extended-release (NVP XR) plus two nucleoside reverse transcriptase inhibitors (NRTIs) due to its excellent safety profile. This observational study examined the demographic and clinical characteristics of HIV-positive patients who switched to dolutegravir plus lamivudine (DTG/3TC) from NVP XR plus 2 NRTIs, following discontinuation of NVP XR from the Canadian market.

**Methods:**

Virally suppressed (< 50 cps/mL) HIV-positive adults ≥18 years who switched from NVP XR plus 2 NRTIs to DTG/3TC between 20 August 2019 and 30 April 2020 were retrospectively identified from Electronic Medical Records housed at Spectrum Health in British Columbia, Canada. Baseline demographic and clinical characteristics were summarized using descriptive statistics at the date of first DTC/3TC prescription. Virologic control, CD4 cell count, weight-related changes and exploratory characteristics related to metabolic syndrome were summarized at baseline and at 12 ± 6-months post-switch using descriptive statistics. Reasons for treatment discontinuation were also captured.

**Results:**

Sixty-nine patients were identified (mean age ± SD = 54.2 ± 8.5 years; 100% male). Mean length of use (±SD) of NVP XR was 4.6 ± 2.6 years. Sixty-three (91.3%) persisted on DTG/3TC at the 12-month timepoint post-switch with 61 (96.8%) virally suppressed < 50 cps/mL and all 63 (100%) virally suppressed < 200 cps/mL. Among persistent patients with CD4 cell counts available, mean CD4 cell count (±SD) remained stable, increasing slightly from 724.4 ± 238.4 to 740.3 ± 240.6 cells/uL. All six (8.7%) patients who discontinued DTG/3TC, discontinued due to tolerability and not effectiveness reasons.

**Conclusion:**

Our findings are the first to examine real-world use of single-tablet, 2-drug DTC/3TC among patients who switched from multi-tablet, 3-drug NVP XR plus 2 NRTIs in Canada. The majority persisted on DTG/3TC and remained virally suppressed at the < 50 cps/mL level 12-months post-switch. This, coupled with excellent tolerability, demonstrates the effectiveness of DTG/3TC in maintaining viral suppression among a unique and stable group of older men.

**Disclosures:**

**Joss de Wet, MBChB CCFP FCFP**, Gilead: Advisor/Consultant|Gilead: Board Member|Gilead: Grant/Research Support|ViiV: Advisor/Consultant|ViiV: Board Member|ViiV: Grant/Research Support **Joann K. Ban, PharmD, MSc**, GlaxoSmithKline Canada Inc.: Employee **Gustavo Verdier, BSc, BPharm, MBA**, GlaxoSmithKline: Stocks/Bonds|ViiV Healthcare ULC: Salary **Juejing Ling, MSc**, GSK: Employee of IQVIA , a company that receives consulting fees from GSK. **Andrean Bunko, MPH**, GlaxoSmithKline Inc.: GSK contracted with IQVIA for this and other projects. I am acting as an author in capacity of my employment with IQVIA. **Michael McKimm, BSc**, ViiV Healthcare: employee.

